# Measurements of cadmium levels in relation to tobacco dependence and as a function of cytisine administration

**DOI:** 10.1038/s41598-024-52234-w

**Published:** 2024-01-22

**Authors:** Karol Wróblewski, Julia Wojnicka, Piotr Tutka, Agnieszka Szmagara, Anna Błażewicz

**Affiliations:** 1 Laboratory of Commercial and Non-Commercial Clinical Trials, University of Rzeszów, Kopisto 2a, 35-959 Rzeszow, Poland; 2 Laboratory for Innovative Research in Pharmacology, University of Rzeszów, Kopisto 2a, 35-959 Rzeszow, Poland; 3 Department of Pathobiochemistry and Interdisciplinary Applications of Ion Chromatography, Chair of Biomedical Sciences, Medical University of Lublin, 1 Chodźki Street, 20-093 Lublin, Poland; 4 Department of Experimental and Clinical Pharmacology, University of Rzeszów, Kopisto 2a, 35-959 Rzeszow, Poland; 5 National Drug and Alcohol Research Centre, University of New South Wales, Sydney, NSW 2031 Australia; 6 Faculty of Medicine, Institute of Biological Sciences, Department of Chemistry, The John Paul II Catholic University of Lublin, Konstantynow 1J, 20-708 Lublin, Poland

**Keywords:** Biomarkers, Medical research

## Abstract

Cigarette smoking delivers a number of heavy metals, including cadmium (Cd), into the body. Bioaccumulation may result in an increase in pathological consequences over time. The assessment of changes in serum Cd concentrations during the treatment of cigarette dependence with cytisine was performed for the first time. Parameters assessing smoking habits, strength of addiction, and effectiveness of therapy were analyzed. Cd was determined before, during, and after the end of treatment. The serum Cd levels were significantly higher in the smokers than in the nonsmokers. Significant differences in Cd concentrations between sampling times in smokers were observed. Individuals who stopped smoking had significantly lower Cd concentrations compared to baseline. A significant positive correlation between the serum Cd before treatment and smoking urges was also obtained. Additionally, salivary Cd determinations were performed before treatment to evaluate the use of this method to assess cigarette addiction. Our findings indicate that Cd can be used as a biomarker of smoking addiction, and provide an alternative assessment of tobacco smoke exposure to other methods. The results provide new knowledge related to Cd concentrations in human body fluids and may play a role in monitoring and assessing the efficacy of cytisine for smoking cessation.

## Introduction

Cadmium (Cd) is a non-essential and toxic metal widely distributed throughout the world. It is classified as a category 1 human carcinogen by the International Agency for Research on Cancer^1^. The presence of cadmium in the human and animal environment, its ability to accumulate in the body, long biological half-life (estimated at 16–38 years in humans), and direct or indirect toxicity, resulting in cell damage pose a serious health risk for humans. Chronic cadmium intoxication can present with chronic renal failure, kidney stones, and in severe cases, secondary skeletal lesions^2^. Inhalation of cadmium fumes strongly disrupts the functioning of the respiratory system. Cd is involved in the development of chronic obstructive pulmonary disease (COPD), idiopathic pulmonary fibrosis (IPF), and acute respiratory distress syndrome (ARDS)^3^.

According to the World Health Organization (WHO), a tolerable weekly intake of cadmium is set at 7 μg/kg/body weight/week. However, research revealed that the metal concentrations at which damage began and at which biochemical changes could be detected may be below those presently considered relatively safe for humans by the WHO^4^. Non-occupational Cd exposure occurs mainly through tobacco smoke followed by dietary sources. The smoking habit constitutes an important source of Cd in the body. Cd concentrations in the total particulate phase of mainstream smoke are higher than most other toxic elements in most tobacco products^5^. Smokers typically have Cd blood and body burdens more than double those of nonsmokers^6^. The average cigarette contains approx. 2 μg Cd (differences may exist by product category). After smoking 1 cigarette, the smoker's lungs receive about 0.1–0.2 μg of Cd^7,8^.

The biological response to exposure to Cd depends on both the level and duration of exposure. Clinical effects may not be present immediately after a single acute, or low-level chronic exposure, however, bioaccumulation may result in an increase in pathological consequences over time. Inhaled Cd accumulates in the lungs, circulates in the bloodstream, and is eliminated from the organism mainly via urine; however, the daily excreted amount represents only about 0.005–0.01% of the total body burden^9^. The largest amounts of Cd are absorbed in the duodenum. Cd is absorbed and transported by a non-specific divalent metal transporter (DMT1) and metal transporter protein 1 (MTP1). Its uptake can also take place via the calcium channels of the transporter system (hZTL1 and ZNT1) responsible for zinc (Zn) transport. Cd is able to replace Zn present in metallothionein, thereby diminishing the ability of Zn to retard oxidative processes^10^. It also easily combines with thiol groups –SH from cysteine or glutathione (GSH), thus bonded to cysteine-rich protein such as metallothionein, causes hepatotoxicity, and then, accumulates in the renal tissue causing nephrotoxicity. In cells Cd is distributed in all organelles, in particular, it binds with proteins in the cytosol, cell nucleus, mitochondrial, and lysosomal membranes^11^.

The mechanisms of Cd toxicity involving Cd-induced oxidative stress and tissue damage are well-known. Cd may contribute to smoking-related lung disease, possibly by altering the redox balance and through macrophage dysfunction^12^. Several studies have demonstrated that the carcinogenic effect of Cd is related to oxidative stress, which can damage all biological macromolecules, while its major consequence is lipid peroxidation shown to be correlated with the exposure levels to Cd^13^. Recently, the incidence of lipid peroxidation has been proposed as a possible mechanism for Cd-induced carcinogenicity. In addition, significant decreases in the gene expressions of antioxidants, detoxification enzymes, and xenobiotic transporters have been reported^14^. It has been shown that Cd may inhibit enzymes involved in damage repair or modification of DNA, activates apoptosis, mutates mtDNA, alters gene expression, inhibits respiratory chain complexes, reduces ATP synthesis, and alters the inner mitochondrial permeability^9^. Moreover, more and more is known about the neurotoxic role of Cd and its exposure which significantly affects energy, amino acid, and lipid metabolism^15^. It is suggested that Cd affects thyroid function^16,17^.

Tobacco use and its associated health consequences including nicotine addiction, respiratory diseases, and cancers are one of the major global health problems. Increases in Cd levels in lung tissue have been correlated with smoking history. The National Health and Nutrition Examination Survey (NHANES) 1999–2018 data in the United States revealed that the accumulation of Cd in the blood due to smoking and passive smoking mediates the increased risk of all-cause mortality^18^.

Biochemical verification of tobacco smoking and abstinence increases scientific rigor and is recommended in clinical trials of smoking cessation^19^. The most commonly used biomarkers of tobacco smoke exposure are carbon monoxide in exhaled air and cotinine, total nicotine equivalents, minor tobacco alkaloids, and 4-(methylnitrosamino)-1-(3-pyridyl)-1-butanol (NNAL) in body fluids. Methods for assessing exposure to tobacco smoke are still being researched and developed^19^.

Effective treatment of nicotine addiction is essential to counteract any health effects associated with exposure to tobacco smoke, including those related to the effects of heavy metals on the human body. Nicotine replacement therapy (NRT), bupropion, varenicline, and the increasingly popular cytisine are recommended for smoking cessation. Cytisine is a plant quinolizidine alkaloid that acts as a partial agonist at nicotinic acetylcholine receptors (nAChRs**)**^20^. Cytisine at standard dosing for smoking cessation is administered for 25 days. The treatment with cytisine is shorter and less expensive than other pharmacological methods, and therefore more accessible to a larger group of smokers^20^. It has been shown that cytisine is more effective than NRT^21^. Recent findings for cytisine compared to varenicline for smoking cessation are mixed^20,22,23^.

The aim of the study was to assess changes in serum Cd levels during the treatment of tobacco addiction with cytisine. The correlation between serum and saliva Cd content in smokers and nonsmokers, the relationship between tobacco smoke exposure as well as addiction strength, and serum and saliva Cd concentrations were also investigated. The possibility of using Cd determination in human body fluids as a method of assessing exposure to tobacco smoke and the strength of tobacco dependence as well as Cd usefulness as a pharmacotherapy control tool, was explored.

## Results

### Characteristics of the sample

A sample of 23 participants was analyzed, including 12 nonsmokers: 9 female (60%), 3 male (37.5%) and 11 active smokers: 6 female (40%), 5 male (62.5%). Two non-smokers and one smoker did not complete the study. The mean age (range) of participants was 29.5 (24.5–40.5) years and 53 (39–57) years in non-smokers and smokers group respectively. The nonsmokers were younger than smokers, while there was no significance between the groups in terms of sex (*p* = 0.4).

### Comparison of two groups of study participants: smokers and non-smokers in terms of Cd concentration in serum and saliva before the treatment

In the first part of the study, the comparison of two groups: smokers and non-smokers in terms of Cd concentrations in serum and saliva before treatment with cytisine (baseline, t_0_) was performed. The results of the Cd determinations are summarized in Table S1 and Table S2. The distribution of the position and variability measures at baseline for Cd in saliva and serum between the participant groups (smokers and non-smokers) and the indication of the significance of the differences between the groups are shown in Table 1. The mean serum concentration of Cd was significantly higher in the smokers' group than in the non-smokers’ group.

**Table 1:** The distribution of the position and variability measures at baseline for Cd in saliva and serum between smokers and non-smokers and the indication of the significance of the differences between both groups of participants.

Measure	Fluid	Distribution within the group				*p*	ƞ^2^
		Non-smokers		Smokers			
		n	Mdn (Q1–Q3)	n	Mdn (Q1–Q3)		
M	Serum	12	0.5 (0.4–0.7)	11	0.90 (0.8–1.0)	**< 0.001**	0.54
	Saliva	7	1.0 (0.6–1.4)	8	0.5 (0.3–0.9)	0.105	0.08
SD	Serum	12	0.1 (0–0.1)	11	0.1 (0.1–0.2)	0.123	0.07
	Saliva	11	0.1 (0–0.4)	10	0.1 (0–0.2)	0.667	− 0.04

M—mean; SD—the standard deviation; Mdn, $\hat{\mu }_{median}$—median; Q1—the first quartile (25%); Q3—the third quartile (75%); p—the *p* value of test; ƞ^2^—eta squared effect size; concentration of Cd was measured in ppb.

Significant values are in bold.

In one participant in the smoking group and one in the non-smoking group, insufficient saliva samples were obtained for analytical testing. Cd was also not detected in 4 and 2 saliva samples obtained from non-smokers and smokers, respectively. Active smokers were characterized by higher mean concentrations of Cd in serum. Measured values of Cd concentrations in seven saliva samples obtained from non-smokers were higher than in the serum. However, in the four other saliva samples analyzed, Cd content was below the limit of quantification. Among the saliva samples, higher concentrations were observed in non-smokers. However, there were no significant differences in the mean Cd concentrations in saliva in both groups of participants.

### Evaluation of the correlation between Cd concentration in saliva and serum (before cytisine treatment)

The correlations between Cd concentration in serum and saliva in the group of non-smokers and smokers at baseline (t_0_) are shown in Table S3. Positive correlations of mean concentrations of Cd between serum and saliva were observed. For standard deviations, a positive relationship was also observed. However, the results do not contain statistically significant correlation coefficients, mainly due to the small sample size and insufficient volumes of saliva samples collected for analytical testing.

### Estimation of between-group differences in means of serum Cd concentration on the first and fourth day of the study

Between-group differences in serum Cd concentration on the 1st day (visit V1, t_1_) and the 4th day of the study (Visit V2, t_4_) are shown in Table 2. On the first day of the study Cd concentration was measured 2, 4, 6, 8, and 10 h after ingestion of the first dose of cytisine. The mean serum concentration of Cd was significantly higher in smokers than in non-smokers.

**Table 2:** Estimation of between-group differences in means of serum Cd concentrations on the first and fourth day of the study.

Visit (sampling time)	Sampling time	Distribution within the group				*p*	ƞ^2^
		Non-smokers		Smokers			
		n	Mdn (Q1–Q3)	n	Mdn (Q1–Q3)		
V1 (t_1_)	2 h	12	0.4 (0.3–0.4)	11	0.6 (0.5–0.8)	0.001	0.41
	4 h	12	0.4 (0.4–0.5)	11	0.7 (0.7–0.8)	< 0.001	0.63
	6 h	12	0.5 (0.4–0.5)	11	0.8 (0.7–0.9)	< 0.001	0.70
	8 h	10	0.4 (0.4–0.5)	11	0.8 (0.7–0.8)	< 0.001	0.62
	10 h	10	0.4 (0.3–0.5)	11	0.8 (0.6–0.8)	0.002	0.45
V2 (t_4_)		10	0.5 (0.4–0.6)	11	0.9 (0.4–1.0)	< 0.001	0.71

Concentration of Cd was measured in ppb.

Cd concentrations at different time points (from 2 to 10 h after the first dose of cytisine) were similar and there was no significant change in Cd levels throughout the day in both smoking and non-smoking participants.

The distributions of Cd concentrations on the 1st day of the study (Visit V1, t_1_) in the 2–10 h range after the first dose cytisine administration for individual participants show Table S4

### Estimation of Cd concentrations within the smoker and non-smoker group before treatment (t_0_), during the treatment (t_1_, t_4_), and after the treatment (t_26_)

**Figure 1:**
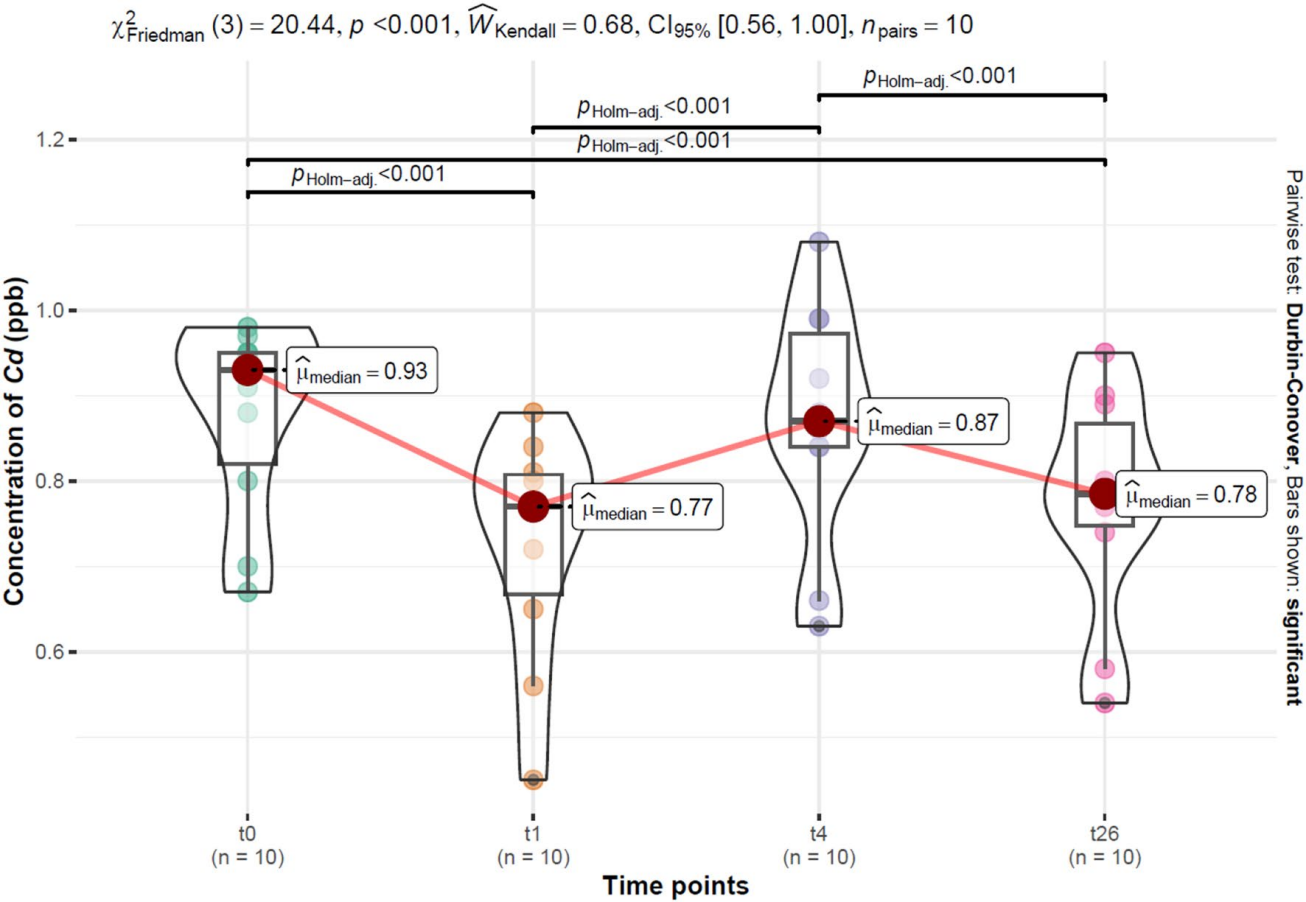
Cd concentration distributions at baseline (t_0_), during treatment (t_1_, t_4_), and after treatment (t_26_), and the results of statistical tests for differences within the group of smokers over time; χ^2^_Friedman_—the Friedman rank sum test statistic; $\hat{W}_{Kendall}$—Kendall's coefficient of concordance; *CI 95%*—confidence interval 95%; $\hat{\mu }_{median}$*—*median;* p*_Holm –adj._—adjusted *p* value with Holm's correction for multiple comparisons.

**Figure 2:**
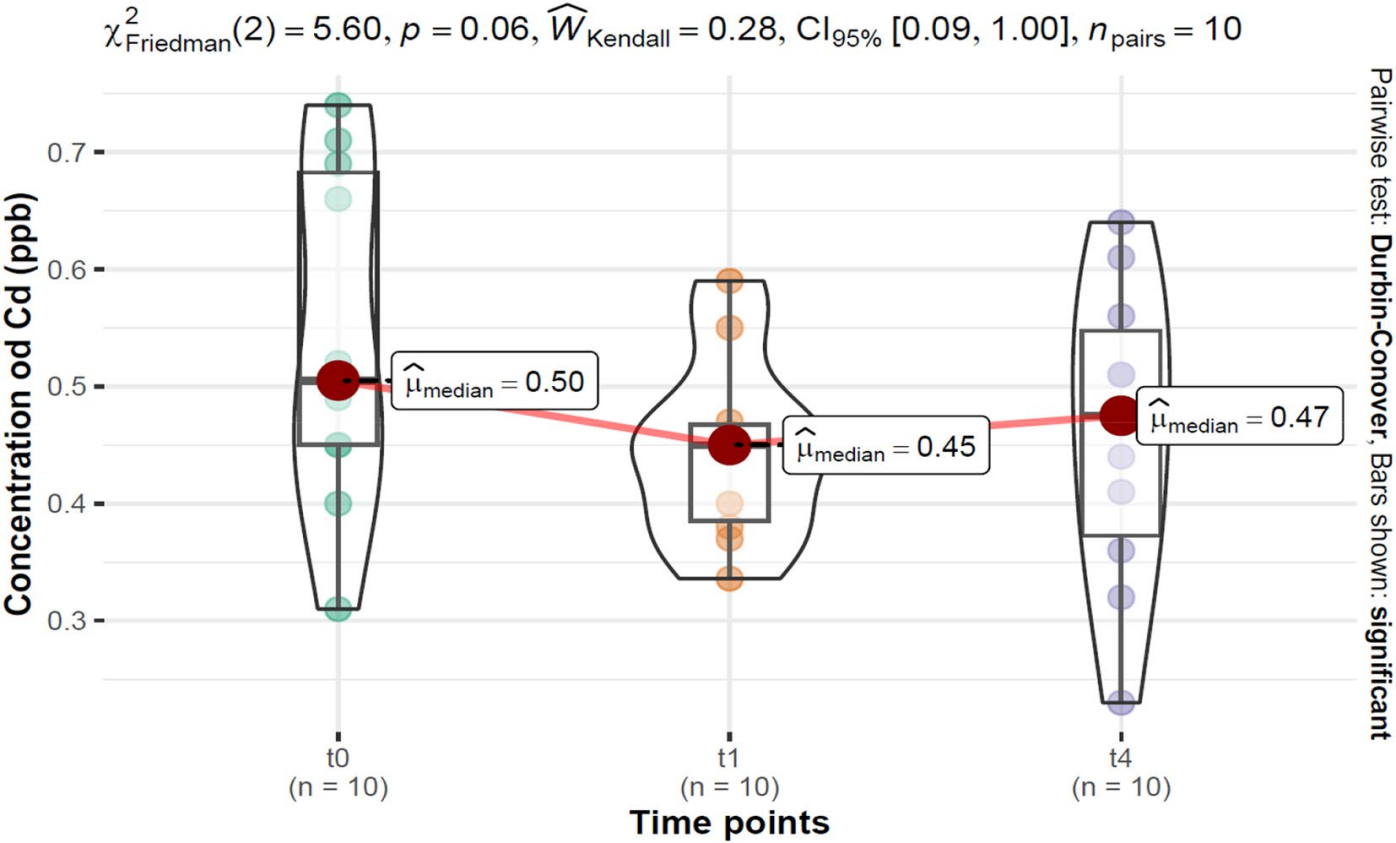
*Cd* concentration distributions at baseline (t_0_), during treatment (t_1_, t_4_), and the results of statistical tests for differences within the group of non-smokers over time; χ^2^_Friedman_—the Friedman rank sum test statistic; $\hat{W}_{Kendall}$—Kendall's coefficient of concordance; *CI 95%*—confidence interval 95%; $\widehat{ \mu }_{median}$*—*median; *p*_Holm-adj._—adjusted *p* value with Holm's correction for multiple comparisons.

A graphical representation of Cd concentration changes with detailed results of the statistical test can be found in Figs. 1 (smokers) and Fig. 2 (non-smokers). Estimation of Cd concentrations carried out within the smoker group before treatment (t_0_), during the treatment (t_1_, t_4_), and after the treatment (t_26_). A significantly lower *Cd* concentration of 0.78 (0.75–0.87) ppb was observed after completion of the treatment, compared with baseline (t_0_), 0.93 (0.82–0.95) ppb. The lowest *Cd* concentration was reached on the 1st day of treatment (t_1_), 0.77 (0.67–0.81) ppb, followed by a concentration of 0.87 (0.74–0.97) ppb at t_4_. Significant differences in *Cd* concentrations were observed between t_0_ and t_1_ (decrease), t_0_ and t_26_ (decrease), t_1_ and t_4_ (increase), t_4_ and t_26_ (decrease).

For comparison, Cd concentrations were examined in non-smokers before treatment (t_0_) and during treatment (t_1_, t_4_). Taking cytisine capsules at the last measuring point (t_4_) resulted is a non-significantly higher *Cd* concentration of 0.48 (0.37–0.55) ppb, compared with baseline (t_0_), 0.50 (0.45–0.68) ppb. The lowest *Cd* concentration was reached on the 1st day of treatment (t_1_), 0.45 (0.38–0.47) ppb. None of the changes in *Cd* concentration between time points were significant.

Of the 10 participants who completed the study, at visit 3 (day 26 of the study), seven patients reported abstinence (which was confirmed biochemically) and three participants had not stopped smoking. The changes in the serum Cd concentrations for the abstinent and non-abstinent groups at visit 3 (t_26_) were therefore examined in comparison with values measured at baseline. The abstinent group showed a significant decrease in the Cd concentration (median at t_0_ = 0.91 ppb, median at t_26_ = 0.77 ppb; *p* = 0.022). The results are presented in Table 3.

**Table 3:** Distributions of Cd concentrations in the group of smokers before (baseline) and after the treatment with estimation of the significance of within-group differences by abstinence factor.

n	Non-abstinent group		*P*	$\hat{r}_{biserial}^{rank}$	n	Abstinent group		*p*	$\hat{r}_{biserial}^{rank}$
	Baseline	After treatment				Baseline	After treatment		
	Mdn (Q1–Q3)	Mdn (Q1–Q3)				Mdn (Q1–Q3)	Mdn (Q1–Q3)		
3	0.95 (0.92–0.96)	0.90 (0.82–0.92)	0.371	1.00	7	0.91 (0.75–0.95)	0.77 (0.68–0.80)	0.022	1.00

$\hat{r}_{biserial}^{rank}$—rank biserial correlation coefficient.

### Determination of the correlations between cytisine and Cd concentration

Determination of the correlations between cytisine and Cd concentrations on the t_1_, t_4_, and t_26_ (smokers) and t_1_, t_4_ (non-smokers) was performed. The results of the correlation study are shown in Table S5. There were no significant correlations between the concentrations of Cd and the cytisine concentration.

### Determination of the correlations between Cd concentration and the number of cigarettes smoked

The results of the correlation study between Cd concentration in serum and saliva before treatment (t_0_) and the number of cigarettes smoked daily according to smoking history are shown in Table 4. The range of cigarettes smoked in the group of active smokers was between 10 and 40 cigarettes per day, *Mdn* = 20, *Q1* = 15, *Q3* = 20. As the number of cigarettes smoked per day increased, an increase in serum Cd concentration was observed (ρ = 0.30). An inverse relationship was found for the measurement of Cd concentration in saliva (ρ = − 0.23). However, in both cases, the results were not statistically significant.

**Table 4:** The correlations between Cd concentration and the number of cigarettes smoked, duration of smoking, the age of smoking initiation, and the age of the participants.

	Sampling time	Fluid	n_pairs_	ρ	*p*		
The correlation between Cd concentration at t_0_ and the number of cigarettes smoked daily according to smoking history	t_0_	Serum	11	0.30	0.367		
		Saliva	8	− 0.23	0.571		
The correlation between Cd concentration at t_0_, t_1_, t_4_, and t_26_, and the number of cigarettes smoked on the previous day	t_0_	Serum	10	0	1.000		
		Saliva	8	0.56	0.149		
	t_1_	Serum	10	0.16	0.620		
	t_4_	Serum	10	0.57	0.083		
	t_26_	Serum	10	0.32	0.478		
The correlation between Cd concentration at t_0_, and the number of cigarettes smoked on 25th day of the study	t_0_	Serum	9	0.39	0.295		
		Saliva	8	0.71	**0.047**		

	Fluid	Duration of smoking			The age of smoking initiation		
		n_pairs_	ρ	*p*	n_pairs_	ρ	*p*
The correlations between the duration of smoking and the age of smoking initiation and serum and saliva Cd concentrations at baseline (t0)	Serum	11	− 0.57	0.066	11	− 0.37	0.265
	Saliva	8	− 0.13	0.754	8	− 0.45	0.269

	Fluid	Group					
		Non-smokers			Smokers		
		n_pairs_	ρ	p	n_pairs_	ρ	p
The correlation between the concentrations of Cd in serum and saliva before treatment and the age of the participants	Serum	12	0.09	0.782	11	− 0.71	**0.014**
	Saliva	7	− 0.21	0.662	8	− 0.36	0.385

Significant values are in bold.

The correlation between Cd concentration in serum and saliva at t_0_, t_1_, t_4_, and t_26_, and the number of cigarettes smoked on the previous day are presented in Table 4. The range of cigarettes smoked the day before treatment in the group of active smokers was between 8 and 25, Mdn = 16.5, Q1 = 12, Q3 = 20. The range of cigarettes smoked on the third day of treatment in the group of active smokers was between 0 and 19, *Mdn* = 7.0, *Q1* = 4, *Q3* = 11. The range of cigarettes smoked on the 25 day of treatment in the group of active smokers was between 0 and 9, *Mdn* = 0, *Q1* = 0, *Q3* = 3. No correlation was found for measured serum Cd concentrations at t_0_ and a slight correlation for determined Cd concentrations at t_1_. In other cases, a more apparent positive correlation was obtained—as the number of cigarettes smoked on the previous day increased, an increase in serum Cd concentration was observed. However, the obtained results were not statistically significant.

The positive correlation between the Cd concentration in serum and saliva at t_0_, and the number of cigarettes smoked on the 25th day of the study was also observed. The correlation was statistically significant in the case of the relationship between Cd levels in saliva and number of cigarettes smoked (ρ = 0.71, *p* = 0.047).

### Determination of the correlation between serum and saliva Cd concentration before treatment and the duration of smoking and the age of smoking initiation

The range of duration of smoking in the group of active smokers was between 5 and 40 years, *Mdn* = 29 years, *Q1* = 17.8 years, *Q3* = 35.8 years. The range of age of smoking initiation in the group of active smokers was between 14 and 24 years, *Mdn* = 18 years, *Q1* = 17 years, *Q3* = 20 years.

The results of the correlation study between serum and saliva Cd concentration before treatment and the duration of smoking and the age of smoking initiation are shown in Table 4. There were no significant correlations between the duration of smoking and the age of smoking initiation and serum and saliva Cd concentrations at baseline (*p* > 0.050).

### Determination of the correlation between the concentrations of Cd in serum and saliva before treatment and the age of the participants

The results of the correlation between the concentrations of Cd in serum and saliva measured before treatment and the age of the participants are shown in Table 4. The group of smokers showed a strong decrease in serum Cd concentration with age, while no such relationship was noted in the non-smoking group. There were no significant correlations between the Cd levels in saliva and the age of the participants in both groups of participants.

### Determination of the correlation between the Cd concentrations in serum and saliva and the therapeutic effect—biochemically confirmed abstinence

The results of the correlation between the Cd concentrations in serum and saliva (measured before the start of treatment and on the 26th day of the study) and the therapeutic effect—biochemically confirmed abstinence (on the 26th day of the study and 6 months after the end of therapy) are shown in Table S6. There was mainly a negative correlation between the measured Cd concentration and the achieved therapeutic effect during cytisine treatment. However, no statistically significant correlations were observed (*p* > 0.050).

### Determination of the correlation between Cd concentrations in serum and saliva and the CO concentration in exhaled air

The results of the correlation between Cd concentrations in serum and saliva and the CO concentration in exhaled air—before the start of treatment (t_0_), on the 4th (t_4_) and 26th day (t_26_) of treatment are shown in Table S7 There were no significant correlations between CO concentration in exhaled air and Cd concentration in serum in the group of smokers measured at baseline, day 4, and day 26 of the study.

### Determination of the correlation between serum and saliva Cd concentrations and QSU-brief test results (nicotine craving assessment)

Ten-item Questionnaire on Smoking Urges (QSU-brief) was used as a measure of craving. The results of the correlation between serum and saliva Cd concentrations (t_0_, t_4_, t_26_) and QSU-brief test results on the 10 h post-dose (visit V1), and on the 4th (visit V2) and 26th day (visit V3) of the study are shown in Table 5. A significant correlation between serum Cd levels and QSU-brief test results on 1st day of the study was obtained (ρ = 0.69, *p* = 0.018).

**Table 5:** Determination of the correlations between Cd concentrations in human fluids and results of QSU-brief and FTND tests.

	Sampling time/time of completing the questionnaire	Fluid	QSU-brief		
			n_pairs_	ρ	*p*
Correlations between Cd concentrations in serum and saliva at baseline and QSU-brief test results	t_0_/visit V1 (10h post-dose)	Serum	11	0.69	**0.018**
		Saliva	8	− 0.15	0.722
	t_4_/visit V2	Serum	10	0.40	0.250
	t_26_/ visit V3	Serum	10	− 0.39	0.267

	Time	Fluid	n_pairs_	ρ	p
Correlations between heavy metal concentrations at baseline, t4, t26 and FTND test score	t_0_	Serum	11	0.05	0.890
	t_4_		11	0.11	0.755
	t_26_		10	0.11	0.760
	t_0_	Saliva	8	− 0.30	0.47

Significant values are in bold.

### Estimation of the correlation between the severity of nicotine dependence, as measured by the FTND test, and the Cd concentrations in serum and saliva

Estimation of the correlation between the severity of nicotine dependence, as measured by the Fagerstrom questionnaire (FTND) and the Cd concentrations in serum and saliva measured at baseline (t_0_), on the 4th day of treatment (t_4_), and after treatment completion (t_26_) are shown in Table 5. There were no significant correlations between Cd concentrations and FTND test results.

## Discussion

Cytisine was used for smoking cessation in this study. It is recommended that smokers quit cigarette smoking on Day 5 (designated target quit day). However, in our research, participants were allowed to stop smoking at any point during the treatment. As therapy progressed, a decrease in the number of cigarettes smoked by participants was observed^24^. It is known, that mean trough cytisine concentrations increased with repeated dosing on the 1st day of the study, and accumulation of cytisine was noted. An increase in the mean plasma concentrations during the first 4 days of the study and decrease in mean trough cytisine concentrations as the study progressed due to a decrease in dosing frequency was observed^25^. The relationship between cytisine levels and therapeutic effect has not been confirmed to date.

It was found that the serum Cd concentrations were significantly higher in smokers than in non-smokers. This finding is consistent with previous studies that showed increased blood Cd concentrations in cigarette smokers^26–29^. However, Cd levels did not exceed toxic values (5 ppb) in any case. Normal blood Cd concentration is less than 5.0 ppb, with most results in the range of 0.5 to 2.0 ppb. Acute toxicity is observed when the blood level exceeds 50 ppb^30,31^. Smokers typically have Cd blood and body burdens more than double those of non-smokers^6^. The blood Cd level in non-smokers is usually lower than 0.5 ppb, while that for current smokers is as high as 1–4 ppb^28,29^. Similar results were obtained in our study, although slightly lower average values were observed for smokers. Mdn values (Q1–Q3) at baseline for non-smokers and smokers were 0.5 (0.4–0.7) ppb and 0.90 (0.8–1.0) ppb, respectively. All determined serum Cd concentrations were below 1 ppb (except one sample of smoker—1.08 ppb, Table S1).

An alternative to serum is the application of saliva for monitoring heavy metal concentrations including in the population exposed to Cd^32–34^. Analysis of heavy metals in saliva can be complementary to that of typical body fluids, such as blood and urine, and may reflect the concentration in other fluids, especially blood^35^. However, according to research conducted by Wang et al., there were no relationships between serum and saliva Cd content^32^. Metal ions are not passively diffused from glands to saliva, but rather actively transported into it. This may explain the lack of correlation between metal levels in saliva and blood^34^. Talio et al.^33^ pointed to the potential usefulness of monitoring Cd levels in saliva as an indicator of smoking addiction. Higher salivary concentrations were recorded in smokers, but the small number of participants (7 total individuals) without appropriate statistical analysis makes the study non-conclusive. Determination of Cd in saliva was also conducted in the present study before the start of treatment with cytisine. In several cases in both the non-smoking and smoking groups, the estimated detection limit (LOD = 0.063 ppb) was not reached. In our study, there were no statistically significant differences between the Cd concentrations in saliva between smokers and non-smokers. Weak positive correlations of the Cd concentrations between serum and saliva were also observed. However, the results are not statistically significant mainly due to the small sample size. Besides Cd can be detected by applying the same technique used for the other matrices. However, compared to serum, we did not demonstrate the usefulness of saliva as a tool for assessing exposure to tobacco smoke and as an indicator of smoking dependence.

Cd accumulates in the body and has a long biological half-life (estimated at 16–38 years in humans)^2^. Blood Cd concentrations reach equilibrium after several months of chronic exposure to Cd which is continuously excreted through the urinary tract. Therefore, blood Cd concentration indicates recent exposure, whereas urine content indicates chronic Cd exposure^36^. For this reason, as confirmed by our study, measuring serum Cd concentrations is a good method for assessing recent changes in Cd exposure. Such a situation occurs during smoking cessation. Significant differences were observed between the Cd serum concentrations measured at baseline (t_0_), during the treatment (t_1_, t_4_), and after the treatment (t_26_) in the group of smokers. The Cd concentration determined after treatment (t_26_) was significantly lower than those obtained at baseline (t_0_). Whereas none of the changes in the Cd concentration between time points were significant in the group of nonsmokers. The results indicate that treatment with cytisine in smokers influences changes in the serum Cd levels, and the effects are already noticeable during a standard 25-day dosing regimen. The 10 participants in the smoking group attended the V3 visit on day 26 of the study the day after they had finished taking cytisine. Seven patients reported abstinence, which was confirmed biochemically by measurement of CO in exhaled air and additionally by detection of serum cotinine. The abstinent group showed a significant decrease in the Cd concentration compared to the measured baseline value (*p* = 0.022). The efficacy of cytisine in the treatment of tobacco dependence has been proven in clinical trials conducted to date^20–23^. The findings of our study indicate that cytisine affects smoking cessation and subsequently Cd levels.

The results presented seem to support the possibility of using Cd as a biomarker of tobacco use and smoking cessation. Biochemical verification of exposure to tobacco smoke plays an important role in determining smoking status, verifying abstinence, monitoring the progress of pharmacotherapy and the effectiveness of treatment. This can be used in clinical trials of smoking cessation as well as in the daily practice of nicotine dependence treatment. Currently, many different biomarkers of smoking are used, which also have their own limitations. Nicotine, cotinine, minor alkaloids, and CO have half-lives from 2 to 16 h. Thus, these biomarkers can be used for smoking detection within the past 1–2 days, depending on the intensity of the smoking level. NNAL can be used for longer-term discrimination and can be detected in the urine for 2 months or longer after stopping tobacco use^19^. Cd measurement, due to its long biological half-life, might be used as a tool not only to assess current smoking status, but also to assess longer-term exposure to Cd. Cd may therefore correlate better with health risks associated with smoking than other markers.

It is known that the strength of addiction correlates with the number of cigarettes smoked. More cigarettes smoked also means higher levels of biomarkers such as cotinine and CO concentration in exhaled air. It was suggested that the stronger the addiction, the lower the chances of success of anti-smoking therapy^37^. It is known that blood Cd concentrations increase with the number of cigarettes smoked per day^26^. Similar relationships can also be seen in our study. However, the many of conducted analyses on the relationship between measured Cd levels and smoking behaviours did not achieve statistical significance. It seems also that a stronger addiction and thus a higher number of cigarettes smoked and a higher Cd concentration before treatment may result in less effective treatment and on average a higher number of cigarettes smoked after treatment. However, the correlation was only statistically significant in the case of the relationship between Cd levels in saliva at baseline and the number of cigarettes smoked on the last day of the study (ρ = 0.71, *p* = 0.047). We also did not obtain a statistically significant relationship between Cd concentrations and the amount of CO in exhaled air in smokers.

FTND^38^ is the most commonly used quantitative measure of nicotine dependence. The higher the score on this test, the higher the level of dependence. Questionaire has also proved successful in predicting the outcome of attempts to stop. Cigarettes per day and time to the first cigarette per day seem to be the most important questionnaire items as indicators of addiction^39^. A second useful test for assessing cigarette dependence is Ten-item Questionnaire on Smoking Urges (QSU-brief)^40^ widely used as a measure of craving. Smoking urges and cravings can be defined as subjective, motivational states responsible for ongoing cigarette use and the inception of relapse. The higher the score on this test, the higher craving. Measuring the strength of urges to smoke could be used to evaluate the severity of cigarette dependence and anticipation of smoking cessation attempts. A rating of the strength of smoking urges can be used as a predictor of quit success^41^. It can be assumed that stronger addiction, which is usually associated with a higher number of cigarettes smoked, should result in higher Cd concentrations in body fluids. However, there were no significant correlations between Cd levels in serum and saliva and FTND test results. Slightly different are the results evaluating the strength of the association between the Cd levels and the score on the QSU-brief. A significant correlation between serum Cd levels at baseline and QSU-brief test results on 1st day of the study was obtained (ρ = 0.69, *p* = 0.018). The stronger the nicotine craving (and therefore the greater the strength of the addiction), the higher the serum Cd concentration.

The outcome of nicotine dependence treatment can be influenced by adherence to dosage recommendations. In the present study, participants completed a study diary on each day of the treatment, in which they recorded the time they took the drug according to the standard dosing schedule. It was found that compliance was adhered to. In addition, serum and salivary cytisine concentrations were measured at each visit and detection of cotinine was performed. Thus, we assume that there were no significant changes in compliance that could have affected the results of the study.

Serum Cd levels, in addition to the efficacy of the tobacco dependence treatment itself, may have been influenced by the different Cd content in cigarettes and other factors that may affect the balance of Cd intake/excretion. Cd levels can therefore also be influenced by factors such as age, medical conditions and additional Cd exposure related to the workplace and food habits. The body burden of Cd increases with age due to its minimal excretion. Cadmium absorption increases in certain physiological conditions, such as iron deficiency and pregnancy^42,43^. However, pregnancy is contraindicated for cytisine treatment and in present study this population was also excluded. The above factors should therefore be taken into account when analysing the results of Cd determinations in body fluids.

## Limitations of the study

In accordance with the results, many of the analyses conducted indicate the existence of possible relationships between Cd content in body fluids and smoking conditions, the strength of cigarette addiction, and the effectiveness of addiction treatment. The existing lack of statistical significance may be related to the small study population, especially as there is literature data supporting the validity of the assumptions made. It is likely that the results could have been statistically significant with a larger sample size. In the present study, many blood sampling was performed (especially on the 1st day of the study—all-day draws performed in the medical center at specific intervals), which caused a significant burden on the patients. It was not possible to recruit more volunteers, as well as this would have been problematic on ethical grounds in this case. In our study, nonsmokers were also younger than smokers. Additional studies are required for arriving at valid conclusions with respect to the incidence of age and sex over exposition level. The influence of medical conditions and the risk of additional Cd exposure (e.g. related to the workplace or food habits) will be the subject of our next work. The use of Cd as a biomarker of tobacco use and abstinence in wider clinical practice requires the establishment of cutoff to distinguish between tobacco users and non-users. The results of the present study may provide a basis for further research, which should be conducted optimally on a larger population.

## Materials and methods

### Chemicals and reagents

Standard of cytisine and Desmoxan® (capsules 1.5 mg) were obtained from Aflofarm (Pabianice, Poland). The standard of cotinine was obtained from Sigma Aldrich (Saint Louis, MO, USA). Methanol, acetonitrile, formic acid (98–100%), ammonium 25%, ammonium formate, hydrochloric acid, and water for LC–MS were purchased from Merck (Darmstadt, Germany). Cd standard solution was purchased from Inorganic Ventures, Christiansburg, Virginia, USA. Certified reference materials (CRMs): EnviroMAT Drinking Water, high (EP-H-2), and EnviroMAT wastewater, high (EU-H-3) were purchased from SCP Science, Quebec, ON, Canada, and NWTM-DWS.2 Canada Environment (the National Water Research Institute, Canada).

### Study design

**Figure 3:**
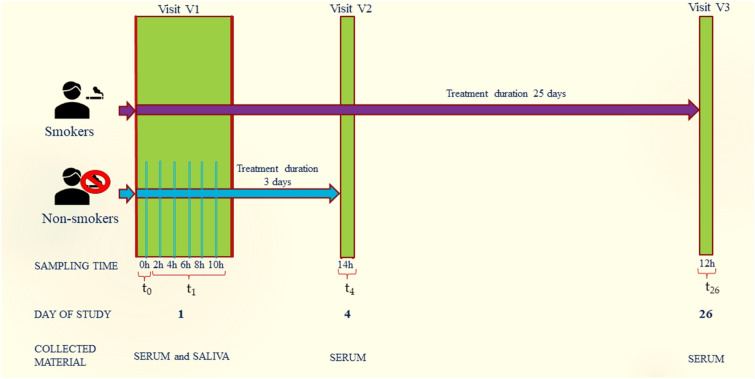
Scheme of the study. On the 1st day (Visit V1) of the study serum and saliva samples for determination of Cd were collected before the start of the treatment (0 h, t_0_) and 2, 4, 6, 8, and 10 h post-first cytisine dose (t_1_). On days 4 and 26 of the study, serum samples were collected 14 and 12 h after the last dose of cytisine on the previous day, respectively.

The present study is a continuation of our earlier research conducted in non-smokers and smokers of conventional cigarettes in order to develop a method for the determination of cytisine in human saliva and serum^44^ and to determine the correlation between cytisine concentration in saliva and serum in non-smokers and smokers as well as to evaluate the safety of cytisine^24^. An additional goal was to determine Cd in the above biological samples and to carry out the analyses being the subject of this study. The participants were divided into two groups: group A—non-smokers and group B—conventional cigarette active smokers. The assessment of nicotine addiction was performed using the Fagerström Test of Nicotine Dependence (FTND)^38^. Smoking urges were measured by Brief Questionnaire on Smoking Urges (QSU-brief)^40^. The status of a smoker has also been confirmed by measuring the concentration of CO in the exhaled air using a smokerlyzer and detection of cotinine by the proprietary LC-QTOF-MS method^44^. Smokers were treated with cytisine according to the 25-day dosing regimen recommended by the manufacturer. Non-smoking participants used cytisine for 3 days at the standard dose. At various time points in the study, biological material (blood and saliva) was collected for analytical tests, and data on the effectiveness and safety of the treatment were collected. Abstinence in participants who quit smoking was verified one day after the end of cytisine therapy (day 26 of the study, visit V3) and 6 months after the drug was discontinued. Participants completed a study diary on each treatment day, in which they recorded the time they took cytisine according to the standard dosing schedule. Any other medication and dietary supplements taken were also recorded.The scheme of the study shows in Fig. 3. The study protocol was approved by the Bioethical Committee of the Medical University of Lublin (approval number KE-0254/165/2018).

#### Inclusion criteria

All participants: 18–65 years of age; signed informed consent. Cigarette smokers: a CO concentration in exhaled breath of at least 10 ppm; smoking at least 10 cigarettes per day over the past 12 months with no period of abstinence > 3 months during that time; motivation to quit smoking.

#### Exclusion criteria

Pregnancy (including those planned during the study) and breastfeeding; myocardial infarction, acute cardiac event, severe arrhythmia, coronary artery bypass (CABG), percutaneous coronary intervention (PCI), stroke, transient ischemic attack in the past 3 months; planned coronary revascularization in next 12 months; cardiac insufficiency (NYHA IV); uncontrolled arterial hypertension (systolic blood pressure > 150 mmHg, diastolic blood pressure > 100 mmHg)**;** psychiatric diseases pharmacologically treated or required hospitalization over the past 5 years; alcohol or drug abuse (except for nicotine) 1 year prior to inclusion in the study; active peptic ulcer disease and/or gastroesophageal reflux disease; pheochromocytoma; hyperthyreosis; liver insufficiency; renal insufficiency; epilepsy; use of smoking cessation medication in the past month; severe allergic response to any drug in the past.

#### Sample size

23 participants were divided into two groups: non-smokers (group A, N = 12), and cigarette smokers (group B, N = 11). Group A included 8 women and 4 men. Group B consisted of 6 women and 5 men.

#### Treatment conditions

Cytisine (Desmoxan, caps. 1.5 mg) was administered orally at doses of 1.5–9 mg per day for 25 days according to the treatment dosage recommended by the manufacturer (group B): day 1–3: 1 capsule every 2 h (6 capsules a day); day 4–12: 1 capsule every 2.5 h (5 capsules a day); day 13–16: 1 capsule every 3 h (4 capsules a day); day 17–20: 1 capsule every 5 h (3 capsules a day); day 21–25: 1 capsule every 10 h (2 capsules a day). Group A was treated with 1 capsule every 2 h (6 capsules a day) for 3 days.

### Determination of Cd in serum and saliva

#### Sample preparation

The serum/saliva samples were dissolved with the acidic treatment in the microwave-assisted digestion system Ethos Up—Advanced Microwave Digestion Labstation (Milestone Srl, Italy) with user-selectable output power (0–1800W with 1W increment). Microwave-assisted acid digestion of samples was performed using a polypropylene rotor equipped with 15 segments with 100 mL capacity, high-pressure Teflon vessels.

The aliquot of 0.25 mL of samples was used for digestion with 3.5 mL of 65% HNO_3_ Suprapur® grade and 1.5 mL of deionized water (DI water, conductivity < 0.08 µS/cm, HLP10 system, Hydrolab, Poland) involved the following steps: the temperature increase to 180 °C in 10 min, the target temperature is constant for 15 min, and cooling step was applied for 15 min. The samples were dissolved with (DI) water up to final volume of 7 mL. Solutions were stored at 4 °C prior to Inductively Coupled Plasma Mass Spectrometry (ICP-MS) measurements.

#### Preparation of standards

The Cd solution (Inorganic Ventures, Christiansburg, Virginia, USA) (10.00 µg/mL in 3.0% HNO_3_ (v/v)) was used as the external calibration standard. The calibration curves were prepared within the range 0.1–100 µg/L by dilution with 6% HNO_3_ Suprapur® grade to 0.1, 1.0, 10, 20, 50 and 100 µg/L. All solutions were prepared in freshly rinsed vials (with 1:1 nitric acid and deionized water (DI) water at least three times). The solution of 6% HNO_3_ was used as the blank solution for drawing the abacus origin of the calibration curve.

#### Measurement of Cd content by ICP-MS

Cd was measured by XSeries 2 ICP-MS (Thermo Scientific, Bremen, Germany) with PlasmaLab software, equipped with a collision/reaction cell operated using 7% H_2_ in He mixture gas (Linde Gaz Polska, Poland), used in conjunction with an ASX-510 autosampler (Cetac, Omaha, Nebraska, USA). Argon (99.9992%, AirProducts, Poland) was used as the plasma generator gas and for sample introduction and nebulization. The instrument was equipped with a concentric nebulizer with low-volume, impact bead Peltier spray chamber, one-piece quartz torch (1.5 mm i.d. injector), simultaneous pulse/analogue detector, and was operated using the following parameters: forward power = 1400 W; nebulizer gas flow = 0.91 L/min; cool gas flow = 13.0 L/min; auxiliary gas flow = 0.80 L/min. Internal standardization was achieved through the simultaneous introduction of 10 ppb solution of yttrium (Yttrium ICP Standard CertiPUR®, Merck, Germany) in 3% of HNO_3_ Suprapur®, using a Y-piece.

After microwave digestion, the samples were diluted in the solution of 6% HNO_3_ in deionized water to reduce non-spectral interferences. Each sample was analyzed in triplicate and FullQuant analysis method was used for the quantification of the data.

The Collision Cell Technology (CCT) with a single H_2_/He cell gas mixture, was used in order to reduce the polyatomic spectral interferences associated with ^55^Mn, such as ^40^Ar^15^N^+^, ^40^Ar^14^N^1^H^+^ and ^54^FeH^+^. The linearity of the calibration curves was evaluated by the respective correlation coefficients (r2) = 0.9999; the LOD of the method was based on 3 × standard deviation of 100 analytical blanks. The accuracy of measurements was checked using certified reference materials (CRMs): EnviroMAT Drinking Water, high (EP-H-2) and EnviroMAT wastewater, high (EU-H-3) (SCP Science, Quebec, ON, Canada), and NWTM-DWS.2 Canada Environment (the National Water Research Institute, Canada). The recoveries were in the range of 95–105%.

### Analysis of cytisine and cotinine in serum and saliva

Determination of cytisine and detection of cotinine (*biomarker* of tobacco smoke exposure) was performed according previously developed LC-ESI-Q-TOF–MS method^44^ using a UHPLC Agilent 1290 Series system (Agilent Technologies, Waldbronn, Germany) equipped with an ESI interface, a 6540 UHD accurate mass Q-TOF detector, and Mass Hunter software for data collection and instrumental control. Quadrupole time-of-flight mass spectrometric analyses were carried out using the electrospray ion source operating in the positive ion mode (ESI+). For sample pre-treatment, the SPE method was applied^44,45^.

### Blood and saliva collection

Blood (5 mL) and saliva (1–2 mL) were collected on 1 day (at least 30 min before and 2, 4, 6, 8, and 10 h after taking the first dose of cytisine), 4 day (14 h after taking the last dose of cytisine at 3rd day) and 26 day (12 h after taking the last dose of cytisine) follow-ups (group B: smokers) or at 1 day (at least 30 min before and 2, 4, 6, 8 and 10 h after taking the first dose of cytisine) and 4 day (14 h after taking the last dose of cytisine at 3rd day) follow-ups (group A: nonsmokers). Ten minutes before collecting saliva, the participants rinsed their mouths three times with a small amount of deionized water. The saliva was collected in sterile plastic containers for 3 min by the spitting method. The collection of saliva was carried out simultaneously with the collection of blood. The biological material was frozen at − 80 °C until analysis.

### Preparation of the serum samples

The collected blood was incubated at room temperature (15–24 °C) until a clot formed (30–40 min). Subsequently, the blood was centrifuged for 15 min at 2000 rpm. The obtained serum was transferred to a sterile plastic tube with an airtight stoper and frozen at − 80 °C.

### Statistical analysis

#### Normality

The normality of the distributions was tested using the Shapiro–Wilk test.

#### Central tendency

Distribution measures of central tendency for numerical variables were expressed in terms of Mdn (Q1–Q3). For categorical variables, the frequencies of each category were expressed both with the percentages per line.

#### Differences between groups

The Mann–Whitney U test was used to compare the means of two independent groups with nonnormal distributions. The effect size measure was estimated using the ƞ^2^ based on the H-statistic: ƞ^2^ (H) = (H − k + 1)/(n − k); where H was the value obtained in the Kruskal–Wallis test; k was the number of groups; n was the total number of observations.

The eta-squared estimate assumed values from 0 to 1 and multiplied by 100 indicates the percentage of variance in the dependent variable explained by the independent variable. Interpretation of effect size was based on Field convention^46^.

#### Dependencies between two nominal variables

The relationships between the two nominal variables were estimated using Fisher's exact test. A measure of the strength of the relationship, phi (φ), was calculated in the case of two dichotomous variables (*df* = 1) and Cramer V in the case of *df* > 1.

#### Correlation

The correlation between the two independent numerical variables was estimated using the Spearman method (ρ). The p values were calculated using the asymptotic t approximation.

#### Differences within groups

The test for within-group mean differences (for dependent data) with more than two groups was performed using the Friedman rank sum test statistic. The effect size measure was estimated using Kendall's concordance coefficient $\hat{W}_{Kendall}$. Interpretation of the effect size was based on the Landis convention^47^.

The significance of differences between groups was calculated using Durbin-Conover post hoc tests. The *p* value was calculated using the Holm correction method for multiple comparisons.

The test for within-group mean differences (for dependent data) with two groups was performed using the Wilcoxon rank sum test. The effect size measure was estimated using rank biserial correlation coefficient, $\hat{r}_{biserial}^{rank}$. Interpretation of the $\hat{r}_{biserial}^{rank}$ effect size was based on the Funder convention^48^.

#### Statistical environment

Analyses were conducted using the R Statistical language (version 4.1.1;^49^ R Core Team 2021) on Windows 10 Pro 64 bit (build 19,044), using the packages effectsize (version 0.8.2;^50^), rstatix (version 0.7.1;^51^), sjPlot (version 2.8.11;^52^), report (version 0.5.1.3;^53^), ggstatsplot (version 0.9.3;^54^), psych (version 2.1.6;^55^), ggplot2 (version 3.4.0;^56^).

### Institutional review board statement

The study was conducted in accordance with the Declaration of Helsinki, and approved by the Ethics Committee of the Medical University of Lublin (approval number KE-0254/165/2018).

### Informed consent statement

Informed consent was obtained from all subjects involved in the study.

## Conclusions

In the present study, Cd concentration in blood serum was measured for the first time during a full standard cycle of cytisine treatment for smoking cessation. The findings of the study indicate that Cd monitoring can be used as an indicator of smoking addiction, and provide an alternative assessment of tobacco smoke exposure to other methods used. Measuring serum Cd concentration may potentially be applied as a method of monitoring and assessing the efficacy of cytisine therapy for smoking cessation. The results may suggest the usefulness of measuring Cd levels before treatment to assess the strength of the addiction and thus also to assess the chances of treatment success. The presented findings open up a valuable area for future research.

### Supplementary Information

Supplementary Information.

## Data Availability

Data are available from the first and the corresponding author upon reasonable request.
