# Supplementary material for: Measurements of cadmium levels in relation to tobacco dependence and as a function of cytisine administration
Source: Sci Rep. 2024 Jan 22;14:1883. doi: 10.1038/s41598-024-52234-w (PMC10803351; doi:10.1038/s41598-024-52234-w)
Supplement: Supplementary file 1 — Supplementary Information. [file 41598_2024_52234_MOESM1_ESM.docx]

# Supplementary Material

**Measurements of cadmium levels in assessing tobacco dependence and in prediction of the efficacy of cytisine in smoking cessation**

Karol Wróblewski^1,2^, Julia Wojnicka^3^, Piotr Tutka^2,4,5^, Agnieszka Szmagara^6^, Anna Błażewicz^3*^

^1^Laboratory of Commercial and Non-Commercial Clinical Trials, University of Rzeszów, Kopisto 2a, 35-959 Rzeszów, Poland; ORCID: 0000-0002-8918-7056 (KW)

^2^Laboratory for Innovative Research in Pharmacology, University of Rzeszów, Kopisto 2a, 35-959 Rzeszów;

ORCID: [0000-0001-5731-4295](https://orcid.org/0000-0001-5731-4295) (PT)

^3^ Department of Pathobiochemistry and Interdisciplinary Applications of Ion Chromatography, Chair of Biomedical Sciences, Medical University of Lublin, 1 Chodźki Street, 20-093 Lublin, Poland; ORCID: 0000-0001-9758-5831 (AB)

^4^Department of Experimental and Clinical Pharmacology, University of Rzeszów, Kopisto 2a, 35-959 Rzeszów, Poland

^5^National Drug and Alcohol Research Centre, University of New South Wales, Sydney, NSW 2031, Australia

^6^The John Paul II Catholic University of Lublin, Faculty of Medicine, Institute of Biological Sciences, Department of Chemistry, Konstantynow 1J, 20-708 Lublin, Poland; ORCID: 0000-0001-9483-058X (AS)

*Corresponding author- [anna.blazewicz@umlub.pl](mailto:anna.blazewicz@umlub.pl)

**Table S1.** Serum Cd concentrations measured for non-smokers and smokers on 1 day (visit V1; at least 30 min before and 2, 4, 6, 8, and 10 h after taking the first dose of cytisine), 4 day (visit V2; 14 h after taking the last dose of cytisine at 3rd day) and 26 day (visit V3; 12 h after taking the last dose of cytisine) follow-ups (group B: smokers) or at 1 day (visit V1; at least 30 min before and 2, 4, 6, 8 and 10 h after taking the first dose of cytisine) and 4 day (Visit V2; 14 h after taking the last dose of cytisine at 3rd day) follow-ups (group A: nonsmokers).

| **Non-smokers** | | | | | **Smokers** | | | | |
| --- | --- | --- | --- | --- | --- | --- | --- | --- | --- |
| **Patient ID** | **Visit** | **Sampling time** | **Cd concentration (ppb)** | **SD** | **Patient ID** | **Visit** | **Sampling time** | **Cd concentration (ppb)** | **SD** |
| 3 | V1 | 0h | 0.400 | 0.030 | 1 | V1 | 0h | 0.950 | 0.100 |
| 3 |  | 2h | 0.330 | 0.060 | 1 |  | 2h | 0.440 | 0.120 |
| 3 |  | 4h | 0.380 | 0.050 | 1 |  | 4h | 0.660 | 0.120 |
| 3 |  | 6h | 0.450 | 0.040 | 1 |  | 6h | 0.520 | 0.140 |
| 3 |  | 8h | 0.380 | 0.090 | 1 |  | 8h | 0.450 | 0.100 |
| 3 |  | 10h | 0.340 | 0.070 | 1 |  | 10h | 0.410 | 0.150 |
| 3 | V2 |  | 0.440 | 0.050 | 1 | V2 |  | 0.840 | 0.200 |
| 5 | V1 | 0h | 0.310 | 0.020 | 1 | V3 |  | 0.950 | 0.050 |
| 5 |  | 2h | 0.330 | 0.070 | 2 | V1 | 0h | 0.700 | 0.230 |
| 5 |  | 4h | 0.380 | 0.090 | 2 |  | 2h | 0.520 | 0.940 |
| 5 |  | 6h | 0.450 | 0.070 | 2 |  | 4h | 0.660 | 0.300 |
| 5 |  | 8h | 0.480 | 0.120 | 2 |  | 6h | 0.700 | 0.090 |
| 5 |  | 10h | 0.560 | 0.040 | 2 |  | 8h | 0.650 | 1.030 |
| 5 | V2 |  | 0.510 | 0.030 | 2 |  | 10h | 0.500 | 0.070 |
| 10 | V1 | 0h | 0.510 | 0.300 | 2 | V2 |  | 0.660 | 0.210 |
| 10 |  | 2h | 0.300 | 0.200 | 2 | V3 |  | 0.540 | 0.180 |
| 10 |  | 4h | 0.360 | 0.150 | 4 | V1 | 0h | 0.630 | 0.400 |
| 10 |  | 6h | 0.450 | 0.180 | 4 |  | 2h | 0.450 | 0.040 |
| 10 |  | 8h | 0.420 | 0.200 | 4 |  | 4h | 0.520 | 0.400 |
| 10 |  | 10h | 0.490 | 0.100 | 4 |  | 6h | 0.660 | 0.070 |
| 13 | V1 | 0h | 0.490 | 0.070 | 4 |  | 8h | 0.650 | 0.220 |
| 13 |  | 2h | 0.360 | 0.020 | 4 |  | 10h | 0.600 | 0.040 |
| 13 |  | 4h | 0.450 | 0.040 | 4 | V2 |  | 0.780 | 0.090 |
| 13 |  | 6h | 0.440 | 0.020 | 6 | V1 | 0h | 0.800 | 0.060 |
| 13 |  | 8h | 0.590 | 0.090 | 6 |  | 2h | 0.710 | 0.055 |
| 13 |  | 10h | 0.510 | 0.070 | 6 |  | 4h | 0.780 | 0.120 |
| 13 | V2 |  | 0.610 | 0.020 | 6 |  | 6h | 0.800 | 0.150 |
| 14 | V1 | 0h | 0.340 | 0.050 | 6 |  | 8h | 0.770 | 0.010 |
| 14 |  | 2h | 0.220 | 0.070 | 6 |  | 10h | 0.730 | 0.200 |
| 14 |  | 4h | 0.280 | 0.220 | 6 | V2 |  | 0.840 | 0.010 |
| 14 |  | 6h | 0.240 | 0.040 | 6 | V3 |  | 0.770 | 0.010 |
| 15 | V1 | 0h | 0.450 | 0.010 | 7 | V1 | 0h | 0.670 | 0.120 |
| 15 |  | 2h | 0.280 | 0.010 | 7 |  | 2h | 0.550 | 0.120 |
| 15 |  | 4h | 0.336 | 0.040 | 7 |  | 4h | 0.580 | 0.140 |
| 15 |  | 6h | 0.330 | 0.120 | 7 |  | 6h | 0.600 | 0.100 |
| 15 |  | 8h | 0.350 | 0.150 | 7 |  | 8h | 0.520 | 0.150 |
| 15 |  | 10h | 0.360 | 0.010 | 7 |  | 10h | 0.560 | 0.210 |
| 15 | V2 |  | 0.320 | 0.010 | 7 | V2 |  | 0.630 | 0.050 |
| 17 | V1 | 0h | 0.450 | 0.010 | 7 | V3 |  | 0.580 | 0.100 |
| 17 |  | 2h | 0.390 | 0.010 | 8 | V1 | 0h | 0.950 | 0.160 |
| 17 |  | 4h | 0.420 | 0.010 | 8 |  | 2h | 0.810 | 0.250 |
| 17 |  | 6h | 0.410 | 0.030 | 8 |  | 4h | 0.890 | 0.040 |
| 17 |  | 8h | 0.400 | 0.010 | 8 |  | 6h | 0.880 | 0.090 |
| 17 |  | 10h | 0.330 | 0.040 | 8 |  | 8h | 0.770 | 0.030 |
| 17 | V2 |  | 0.230 | 0.080 | 8 |  | 10h | 0.790 | 0.050 |
| 19 | V1 | 0h | 0.660 | 0.030 | 8 | V2 |  | 0.920 | 0.180 |
| 19 |  | 2h | 0.400 | 0.010 | 8 | V3 |  | 0.890 | 0.030 |
| 19 |  | 4h | 0.360 | 0.020 | 9 | V1 | 0h | 0.910 | 0.020 |
| 19 |  | 6h | 0.390 | 0.300 | 9 |  | 2h | 0.800 | 1.200 |
| 19 |  | 8h | 0.350 | 0.200 | 9 |  | 4h | 0.840 | 0.900 |
| 19 |  | 10h | 0.370 | 0.150 | 9 |  | 6h | 0.880 | 2.200 |
| 19 | V2 |  | 0.560 | 0.010 | 9 |  | 8h | 0.940 | 1.500 |
| 20 | V1 | 0h | 0.710 | 0.200 | 9 |  | 10h | 0.900 | 1.900 |
| 20 |  | 2h | 0.550 | 0.100 | 9 | V2 |  | 0.880 | 0.050 |
| 20 |  | 4h | 0.620 | 0.120 | 9 | V3 |  | 0.800 | 0.010 |
| 20 |  | 6h | 0.580 | 0.040 | 11 | V1 | 0h | 0.950 | 0.230 |
| 20 |  | 10h | 0.600 | 0.130 | 11 |  | 2h | 0.590 | 0.050 |
| 20 | V2 |  | 0.640 | 0.180 | 11 |  | 6h | 0.660 | 0.030 |
| 21 | V1 | 0h | 0.520 | 0.125 | 11 |  | 8h | 0.780 | 0.050 |
| 21 |  | 2h | 0.390 | 0.335 | 11 |  | 10h | 0.800 | 0.050 |
| 21 |  | 4h | 0.480 | 0.185 | 11 | V2 |  | 0.860 | 0.120 |
| 21 |  | 6h | 0.510 | 0.325 | 11 | V3 |  | 0.770 | 0.040 |
| 21 |  | 8h | 0.440 | 0.090 | 12 | V1 | 0h | 0.970 | 0.070 |
| 21 | V2 |  | 0.360 | 0.050 | 12 |  | 2h | 0.680 | 0.200 |
| 22 | V1 | 0h | 0.690 | 0.135 | 12 |  | 4h | 0.700 | 0.900 |
| 22 |  | 2h | 0.610 | 0.235 | 12 |  | 6h | 0.880 | 0.200 |
| 22 |  | 4h | 0.550 | 0.145 | 12 |  | 8h | 0.800 | 0.150 |
| 22 |  | 6h | 0.470 | 0.255 | 12 |  | 10h | 0.890 | 0.090 |
| 22 |  | 8h | 0.430 | 0.050 | 12 | V2 |  | 1.080 | 0.010 |
| 22 |  | 10h | 0.330 | 0.050 | 12 | V3 |  | 0.900 | 0.020 |
| 22 | V2 |  | 0.410 | 0.225 | 16 | V1 | 0h | 0.880 | 0.120 |
| 24 | V1 | 0h | 0.740 | 0.050 | 16 |  | 2h | 0.560 | 0.010 |
| 24 |  | 2h | 0.660 | 0.275 | 16 |  | 4h | 0.700 | 0.070 |
| 24 |  | 4h | 0.550 | 0.165 | 16 |  | 6h | 0.880 | 0.120 |
| 24 |  | 6h | 0.580 | 0.380 | 16 |  | 8h | 0.800 | 0.100 |
| 24 |  | 8h | 0.520 | 0.050 | 16 |  | 10h | 0.770 | 0.130 |
| 24 |  | 10h | 0.530 | 0.060 | 16 | V2 |  | 0.990 | 0.036 |
| 24 | V2 |  | 0.510 | 0.050 | 16 | V3 |  | 0.740 | 0.070 |
|  |  |  |  |  | 23 | V1 | 0h | 0.980 | 0.050 |
|  |  |  |  |  | 23 |  | 2h | 0.800 | 0.225 |
|  |  |  |  |  | 23 |  | 4h | 0.840 | 0.135 |
|  |  |  |  |  | 23 |  | 6h | 0.840 | 0.425 |
|  |  |  |  |  | 23 |  | 8h | 0.740 | 0.210 |
|  |  |  |  |  | 23 |  | 10h | 0.880 | 0.345 |
|  |  |  |  |  | 23 | V2 |  | 0.990 | 0.050 |
|  |  |  |  |  | 23 | V3 |  | 0.800 | 0.050 |

SD- standard deviation

**Table S2.** Saliva Cd concentrations measured for non-smokers and smokers.

| **Non-smokers** | | | **Smokers** | | |
| --- | --- | --- | --- | --- | --- |
| **Patient ID** | **Cd concentration (ppb)** | **SD** | **Patient ID** | **Cd concentration (ppb)** | **SD** |
| 3 | 0.980 | 0.700 | 1 | 0.933 | 0.162 |
| 5 | <LOD | 0.000 | 2 | 0.933 | 0.808 |
| 10 | * |  | 4 | * |  |
| 13 | 8.307 | 2.125 | 6 | 0.560 | 0.485 |
| 14 | 0.267 | 0.083 | 7 | 0.345 | 0.123 |
| 15 | <LOD | 0.000 | 8 | 0.284 | 0.118 |
| 17 | <LOD | 0.000 | 9 | 0.165 | 0.098 |
| 19 | 0.692 | 0.236 | 11 | 0.000 | 0.000 |
| 20 | 0.457 | 0.178 | 12 | 1.045 | 0.035 |
| 21 | 1.307 | 0.566 | 16 | <LOD | 0.000 |
| 22 | 1.556 | 0.135 | 23 | <LOD | 0.000 |
| 24 | <LOD | 0.000 |  |  |  |

LOD -limit of detection

*Insufficient saliva samples obtained for analytical test

**Table S3.** The correlations between Cd concentration in serum and saliva in the group of non-smokers and smokers at baseline.

| **Non-smokers** | | | | **Smokers** | | | |
| --- | --- | --- | --- | --- | --- | --- | --- |
| **Measure** | **n_pairs_** | **ρ** | **p** | **Measure** | **n_pairs_** | **ρ** | **p** |
| M | 7 | 0.11 | 0.840 | M | 8 | 0.12 | 0.772 |
| SD | 11 | 0.51 | 0.107 | SD | 10 | 0.12 | 0.734 |

*n_pairs_* – number of pairs; ρ – the correlation coefficient estimated by Pearson method

**Table S4.** The distributions of Cd concentrations on the first day of the study (Visit V1) in the 2-10 h range after the first dose cytisine administration for individual participants.

| **Cd serum concentration (ppb): mean value (range)** | | | |
| --- | --- | --- | --- |
| **Patient ID** | **Non-smokers** | **Patient ID** | **Smokers** |
| 3 | 0.38 (0.34 – 0.38) | 1 | 0.45 (0.44 – 0.52) |
| 5 | 0.45 (0.38 – 0.48) | 2 | 0.65 (0.52 – 0.66) |
| 10 | 0.42 (0.36 – 0.45) | 4 | 0.60 (0.52 – 0.65) |
| 13 | 0.45 (0.44 – 0.51) | 6 | 0.77 (0.73 – 0.78) |
| 14 | 0.24 (0.23 – 0.26) | 7 | 0.56 (0.55 – 0.58) |
| 15 | 0.34 (0.33 – 0.35) | 8 | 0.81 (0.79 – 0.88) |
| 17 | 0.40 (0.39 – 0.41) | 9 | 0.88 (0.84 – 0.90) |
| 19 | 0.37 (0.36 – 0.39) | 11 | 0.72 (0.64 – 0.79) |
| 20 | 0.59 (0.57 – 0.61) | 12 | 0.80 (0.70 – 0.88) |
| 21 | 0.46 (0.43 – 0.49) | 16 | 0.77 (0.70 – 0.80) |
| 22 | 0.47 (0.43 – 0.55) | 23 | 0.84 (0.80 – 0.84) |
| 24 | 0.55 (0.53 – 0.58) |  |  |

**Table S5.** Determination of correlations between cytisine and Cd concentration.

| **Group** | **Sampling time** | **n_pairs_** | **ρ** | **p** |
| --- | --- | --- | --- | --- |
| non-smokers | t1 | 12 | 0.00 | 0.991 |
|  | t4 | 10 | -0.21 | 0.555 |
| smokers | t1 | 11 | 0.17 | 0.620 |
|  | t4 | 10 | 0.57 | 0.083 |
|  | t26 | 7 | 0.32 | 0.478 |

**Table S6**. Determination of correlations between the therapeutic effect - biochemically confirmed abstinence on the 26th day of the study (visit V3), 6 months after the end of therapy, and serum and saliva heavy metal concentrations at baseline (t_0_) and 26th (t_26_) day of the study.

| **Sampling time** | **Fluid** | **Abstinence on the 26th day**  **of the study** | | | **Abstinence 6 months**  **after the end of therapy** | | |
| --- | --- | --- | --- | --- | --- | --- | --- |
|  |  | n_pairs_ | 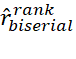 | p | n_pairs_ | 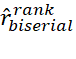 | p |
| t_0_ | Serum | 10 | -0.26 | 0.452 | 4 | -0.77 | 0.225 |
|  | Saliva | 8 | -0.70 | 0.055 | 4 | 0.26 | 0.742 |
| t_26_ | Serum | 10 | -0.42 | 0.226 | 4 | -0.77 | 0.225 |

**Table S7.** Determination of the correlations between Cd concentrations in serum and saliva and the CO concentration in exhaled air at baseline*.*

| **Sampling time** | **Fluid** | **Smokers** | | |
| --- | --- | --- | --- | --- |
|  |  | ***n_pairs_*** | **ρ** | ***p*** |
| t_0_ | Serum | 12 | 0.26 | 0.428 |
|  | Saliva | 8 | 0.17 | 0.690 |
| t_4_ | Serum | 10 | 0.08 | 0.826 |
| t_26_ | Serum | 10 | 0.24 | 0.500 |
